# Factors Influencing the Popularity of Artificial Insemination of Mares in Europe †

**DOI:** 10.3390/ani9070460

**Published:** 2019-07-19

**Authors:** Alicja Kowalczyk, Ewa Czerniawska-Piątkowska, Marian Kuczaj

**Affiliations:** 1 Department of Environment, Animal Hygiene and Welfare, Wrocław University Of Environmental and Life Sciences, Chełmońskiego 38C, 51-630 Wrocław, Poland; 2 Department of Ruminant Science, West Pomeranian University of Technology, Klemensa Janickiego 29, 71-270 Szczecin, Poland; 3 Institute of Animal Breeding, Wrocław University Of Environmental and Life Sciences, Chełmońskiego 38C, 51-630 Wrocław, Poland

**Keywords:** equine, insemination, semen, stallion

## Abstract

**Simple Summary:**

The popularity of mare insemination as an element affecting the dynamic growth of breeding progress among horses in Europe is subject to various fluctuations. The success of this method of reproduction should be considered first of all in terms of the quality of semen available on the market, the types of semen storage technology, the profitability of its use resulting from the effectiveness of insemination with the selected type of semen, and factors affecting the success of artificial insemination. The purpose of this work was to present the factors determining the popularity of artificial insemination of mares in Europe. The presented statistics show that the popularity of the use of chilled semen has gradually increased in the group of sport mares, while in the group of breeding mares, the popularity of frozen semen has increased. In the remaining group of mares (not classified as sport or breeding), insemination with chilled semen was dominant. To talk about the success of artificial insemination of horses in Europe, it is necessary to look thoroughly at these aspects that affect the popularity of this reproduction biotechnology, and in particular to improve the quality of insemination doses.

**Abstract:**

The purpose of this review was to analyze factors affecting the popularity of artificial insemination of mares in Europe in the context of sperm quality. Taking into account the prices of stallion semen on the world market, efficiency is important for the profitability of its use in artificial insemination programs in Europe. To increase the efficiency of a semen insemination facility, it is necessary to correctly and objectively assess the quality of semen. The available range of tools allows an effective evaluation of the potential fertility of a stallion. For several years, artificial insemination programs in Europe have been gaining popularity. However, the frequency of chilled or frozen semen use is still quite low. This is mainly due to the common, negative opinion about the effectiveness of the use of packaged insemination doses as opposed to natural insemination. Unfortunately, the quality of the semen offered often deviates from expectations, which results in unsatisfactory (and therefore unprofitable) pregnancy rates. This review presents the popularity structure of chilled and frozen semen use in European horse breeding as well as the current state of research on the effectiveness of semen production technology. It is shown that the popularity of using chilled semen in the artificial insemination of mares in Europe has been gradually increasing in the group of sport mares, while in the group of breeding mares, in recent years, frozen semen has been gaining popularity. In the remaining group of mares (not classified as sport or breeding), insemination with chilled semen has been dominant.

## 1. Introduction

Horse breeding in Europe is mainly based on the selection of horses in terms of sport and breeding performance. To a much lesser extent, horses are selected for recreational use. The basic assumption of horse reproduction in Europe is profitability, which results from the sale of a horse or its lease. In Europe, the selection of horses for breeding is made based on the pedigree, sporting results, and temperament of the animal concerned. The decisive factor when choosing a sire is the price of its semen.

Artificial insemination is widely used in modern animal reproduction [1], especially when genetic improvement is taken into account. The percentage of foals coming into the world as a result of insemination with chilled or frozen semen has reached about 90% [2]; in Europe, it is only 45% [3]. Methods of semen freezing, the composition of extenders for stallion sperm storage (necessary to maintain the integrity of cytoplasmic membranes and fertilizing potential), insemination time, insemination site, and the optimal insemination dose are the factors contributing to the variability of pregnancy indices [4]. To minimize this variability, scientists and practitioners try to develop optimal, standardized protocols both for sperm freezing and for insemination. Research is also aimed at predicting the fertility of spermatozoa subjected to freezing and thawing. In particular, the attempts are focused on the identification of specific molecular markers that can potentially be highly correlated with fertility, and thus can increase the susceptibility to freezing and the effectiveness of artificial insemination with stallion semen. The aim of this work is to analyze factors affecting the popularity of different types of stallion sperm (chilled and frozen) in Europe.

### The Structure of Chilled and Frozen Semen Use for Artificial Insemination in Europe.

As mentioned in the introduction, mares artificially inseminated in Europe constitute fewer than half of those intended for reproduction. Table 1 presents data concerning the structure of mare insemination in Europe in the period 2013–2017 (data come from insemination stations; they were collected and archived based on private summaries from three leading breeding stations in northern France (*N* = 986), south-western Poland(*N* = 554), and eastern Germany (*N* = 940)).

Artificial insemination is used predominantly in sport mares and has been increasingly popular since 2013. In 2017, sport mares accounted for 67% of all mares inseminated at the breeding stations. In the case of breeding mares, a slight decrease in the use of artificial insemination as the main breeding technique has been observed. The percentage of artificially inseminated mares decreased from 37% in 2013 to 29% in 2017. No significant variability in the frequency of artificial insemination among breeding mares has been observed in particular years. The other mares (not classified as sport or typically breeding ones) constitute a small percentage of all artificially inseminated mares, and the popularity of this kind of insemination among this group has been decreasing significantly since 2013.

**Figure 1:**
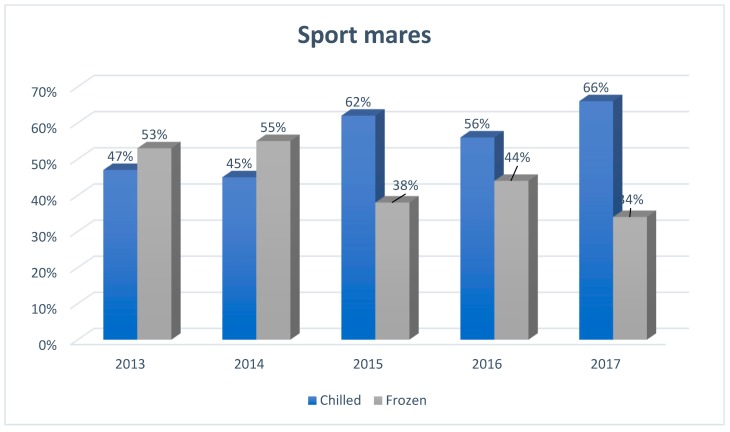
The structure of insemination of sport mares in Europe, including the division into the type of semen used for artificial insemination (*N* = 2480), developed based on reports from European insemination stations.

**Figure 2:**
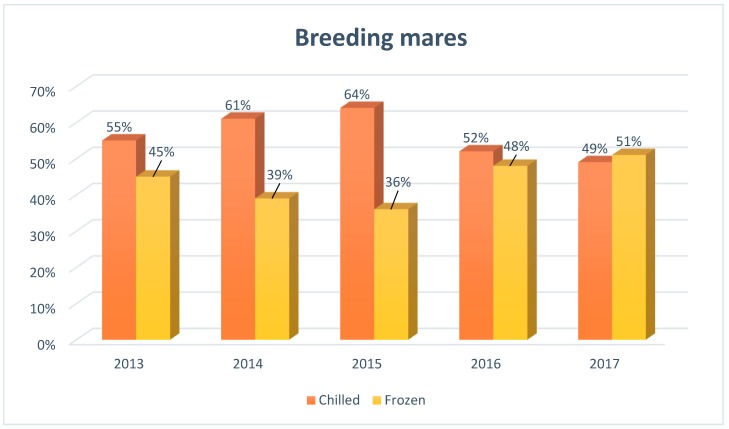
The structure of insemination of breeding mares in Europe, including the division into the type of semen used for artificial insemination (*N* = 2480), developed based on reports from European insemination stations.

**Figure 3:**
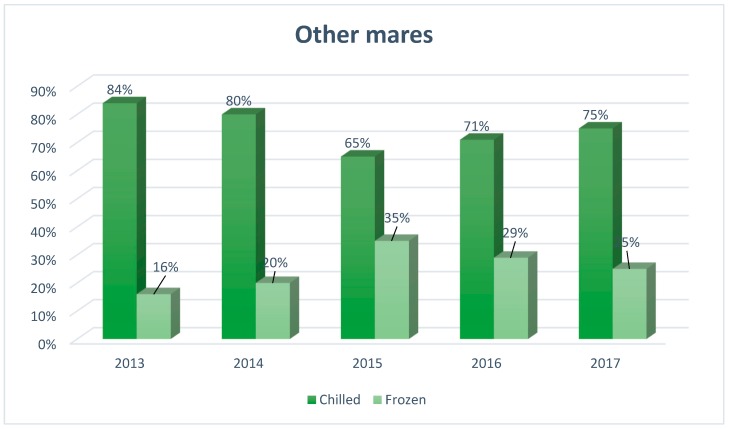
The structure of insemination of other mares in Europe, including the division into the type of semen used for artificial insemination (*N*= 2480), developed based on reports from European insemination stations.

Figure 1, Figure 2 and Figure 3 show the popularity of semen used in mares breeding in Europe from 2013 to 2017, taking into account the type of semen used for this purpose (chilled, frozen).

In 2013 and 2014, the popularity of using chilled semen was lower than frozen, while from 2015 its popularity began to grow dynamically (by about 15% compared to previous years). This popularity reached 62% of inseminated sport mares and was 24% higher than in the case of frozen semen. In subsequent years (2016 and 2017), it remained at a similar level, still dominating over frozen semen. 

Initially, in the period of 2013–2015, the use of chilled semen in the artificial insemination of breeding mares significantly prevailed over frozen, representing 55% (2013), 61% (2014) and 64% (2015) of all breeding mares submitted to insemination in the European insemination centers. However, since 2016, there has been a significant increase in the frequency of the use of frozen semen, reaching a similar level as the use of chilled semen.

In the other mares, the use of frozen semen constitutes a small percentage, which is on average about 25% of all mares in this group. Since 2015, the use of chilled semen is significantly more popular than frozen, although in recent years (2015, 65%; 2016, 71%; 2017, 75%) its popularity has slightly decreased.

## 2. Semen Evaluation

In Europe, only a few laboratories in stallion semen production stations routinely use flow cytometry to examine sperm quality. The other stations use the conventional method, i.e., the determination of the percentage of pregnancies achieved among mated/inseminated mares. The use of cytometric methods to improve the quality of biological value of stallion ejaculate evaluation is not a routine procedure in Europe, and the data obtained in the area of individual stallion fertilization effectiveness are quite limited. As demonstrated in the latest research, it is possible to obtain a high level of stallion fertility prediction by (partly) introducing cytometric analysis as the basic method of semen evaluation and by establishing a new protocol for semen evaluation [5]. By combining microscopic observations, computerized motility analysis and flow cytometry, objective and practical tools to estimate a stallion’s fertility can be provided. The evaluation of stallion semen before its application in breeding and artificial insemination (AI) is crucial in the case of breeding horses. Failure to eliminate poor quality ejaculates may result in poor fertility rates, and this is the most important issue in the evaluation of a stallion’s reproductive performance. Gottschalk et al. [6] indicates the relationship between the fertility of stallions and semen traits. The indices of semen quality, including progressive movement, complete sperm motility and morphology, may explain some fluctuations in a stallion’s fertility [7,8,9]. The fertility of stallions should be measured by the degree of pregnancies per cycle [2,10,11] or non-return rate [12,13].

## 3. Semen Storage

Cryopreservation is currently the only possible method of sperm storage for an indefinite period. However, the cryopreservation and thawing processes reduce sperm viability and motility in all studied farm animal species, including horses [14,15,16]. Also, there is evidence that cryopreservation leads to DNA defragmentation, which is important in terms of fertilization and normal embryonic development [17]. Many of the harmful effects resulting from cryopreservation can be attributed to osmotic stress. The formation of extracellular ice crystals starts during cooling below the temperature of 0 °C. This, in turn, causes a large increase in osmolarity, which exposes sperm cells to extreme osmotic stress [18]. Also, cryoprotectants produce hyperosmotic cryodiluent, which causes cell dehydration through osmosis. Since dehydration is essential for maintaining sperm viability after thawing, extreme hyperosmolarity induces cellular stress (water flows through the sperm membrane to the water channels, attempting to balance osmolarity) [19].The result of these osmotic stressors is damage to the cytoplasmic membranes [20], DNA damage [21] and the production of reactive oxygen species (ROS) [22]. However, besides the possibility of long-term storage of stallion semen, chilled semen is also used in Europe. In this case, the semen is usually processed using commercial passive cooling devices that slowly cool the diluted semen to about 5 °C, a temperature that is low enough to limit the cell metabolism to a sufficient degree to maintain the function of the semen up to about 72 h after its collection. However, stallion spermatozoa are much more susceptible to cold shock than the sperm of other species. As in the case of cryopreservation, there are numerous unexplained differences between stallions in the suitability of their semen for storage at low temperatures [23].

## 4. Time of Insemination

Bearing in mind the superior breeding goal (a horse with high sport value), European horse breeders, from an economic point of view, desire the horses to be born in early spring. This is because horses competing in sport arenas born in the same year, but at intervals of a few months, will be characterized by a significantly different degree of training and differences in body composition and development. Considering that a sport horse is supposed to achieve the desired results in a selected competition, the season of birth is of great importance in the context of acquired skills and experience. The time when a foal is born is, therefore, an important economic factor. Another important timing factor for breeding profitability is insemination time, since both too early and too late sperm deposition in the female reproductive tract is not only a reason for losing valuable time, but also a reason for losing funds invested in the purchase of semen and the preparation of mare insemination. Insemination with cryopreserved semen should take place at a time similar to ovulation; specific protocols developed to coordinate the insemination time with ovulation induction yielded promising results. Samper showed that most mares (94%) ovulate between 36 and 42 h after ovulation induction using human chorionic gonadotrophin (hCG) or deslorelin (if the mare is in oestrus, and the dominant preovulatory follicle and endometrial oedema are present at the time) [24]. There are, however, conflicting theories about the optimal time of insemination in relation to ovulation itself. Many research laboratories have examined pregnancy indices concerning mare insemination before and after ovulation. Some researchers argue for the insemination of mares both before and after ovulation. They recommend the insemination of mares at 24 and 40 h after ovulation induction, while others recommend it at 36 h and then again between 42 and 44 h after ovulation induction. For both schemes, it is assumed that ovulation occurs between two inseminations. Other researchers say that only one insemination is needed for acceptable pregnancy rates. Barbacini et al. [25] evaluated insemination protocols and found no difference in pregnancy rates between mares inseminated at 24 and 40 h after hCG administration (46%), (ovulation likely occurred between 36 and 42 h) and the pregnancy rate in mares which were inseminated only once after ovulation induction (47%). Single insemination after ovulation provided similar pregnancy rates per cycle, as reported by Hemberg et al. [26] (45.4%) and Metcalf [27] (47%). From an economic point of view, the most profitable option for the breeder is the induction and monitoring of ovulation, insemination at the time most similar to ovulation, and simultaneous use of the smallest possible number of semen doses. Considering that the majority of stallion stations in Europe offer single portions of semen (one straw of 0.5 mL of frozen semen or one vial of 20 mL of chilled semen) in the price range from €450 to €3500, it becomes clear that the less semen used per effective insemination, the lower the cost of one pregnancy.

## 5. Reduction in the Number of Spermatozoa

A good effectiveness of fertilization can be achieved by using frozen/thawed semen with reduced sperm concentration, using insemination near the follicle. Recently, research has focused on deep insemination (to uterine horns) or hysteroscopy. Although hysteroscopic insemination was originally aimed at improving pregnancy rates in mares inseminated by low fertility stallions [28], this technique did not yield the desired effects in this case [29]. Indicators of pregnancy after hysteroscopic or deep insemination using a small concentration of sperm are very diverse for frozen/thawed semen. Lindsey et al. [30] found a 37.5% pregnancy rate in semen inseminated with sperm containing 15,106 active frozen/thawed spermatozoa inseminated with hysteroscopy, while Morris et al. [31] found a higher prevalence rate of 64.3% (9/14) after insemination with a dose of 14,106 motile, frozen/thawed sperm. The author obtained the same prevalence of pregnancy when mares were inseminated with a dose of 14,106 motile, frozen/thawed sperm to the uterine body (66.7%, 8/12). Petersen et al. [32] reported 64% (7/11) pregnancies among mares inseminated with a dose of 50,106 frozen/thawed, progressively motile spermatozoa at 12 and 24 h after hCG administration, using deep insemination to the uterine horn. When the same mares were inseminated with a dose of 500,106 sperm cells to the uterus, only 4/11 mares became pregnant (37%).

## 6. Conclusion

The analysis of stallion sperm is an important part of reproductive performance evaluation. The results of semen evaluation cannot be interpreted without a thorough knowledge of mares and of the management effects of the herd that could play a role in or affect the potential fertility of the stallion. Such an evaluation should be carried out using modern tools such as flow cytometry, allowing objective, fast and simultaneous analysis of many quality parameters while analyzing a large number of spermatozoa. Bearing in mind the development of artificial insemination in Europe, it is important to popularize preserved semen by improving its quality and its effectiveness of use in insemination.

The popularity of using chilled semen in the artificial insemination of mares in Europe has been gradually increasing in the group of sport mares, while in the group of breeding mares, in recent years, frozen semen has been gaining popularity. In the remaining group of mares (not classified as sport or breeding), insemination with chilled semen has been dominant. Likely, the popularity of the type of semen used for insemination is primarily determined by the effectiveness of insemination with the chosen type of dose (chilled or frozen) and the biological quality of the ejaculate stored in a liquid or frozen state, which influences this effectiveness.

The main purpose of an artificial insemination program is to determine the most effective use of semen without lowering the rate of pregnancy obtained. Insufficient sperm quality, whether chilled or frozen, directly affects the popularity of its use, affecting breeders’ profit levels (not only for the owner of the mares, but also for the owner of the stallion/producer of the semen).

## Figures and Tables

**Table 1:** The structure of mare insemination in Europe, including the division into their type of use (*N* = 2480), developed based on reports from European insemination stations.

Years	Mares		
	Sport	Breeding	Other
2013	49%	37%	14%
2014	53%	40%	7%
2015	58%	33%	9%
2016	61%	37%	2%
2017	67%	29%	4%
